# Multiple osteochondromas (MO) in the forearm: a 12-year single-centre experience

**DOI:** 10.1007/s11751-016-0267-1

**Published:** 2016-10-13

**Authors:** John Ham, Mark Flipsen, Marianne Koolen, Arnard van der Zwan, Konrad Mader

**Affiliations:** 1 Orthopædic Department, Expertise Center MO, OLVG, Amsterdam, The Netherlands; 2 Section Upper Extremity, Department of Orthopaedic, Trauma and Spine Surgery, Asklepios Klinik Altona, 22763 Hamburg, Germany; 3 Berliner Freiheit 9, Aalto- Hochhaus 14/9, 28327 Bremen, Germany

**Keywords:** Multiple hereditary exostoses (MHE), Multiple osteochondromas (MO), Masada classification, Forearm reconstruction, Corrective osteotomy, External fixation, Review

## Abstract

Multiple osteochondromas (MO) are a rare autosomal dominant disorder characterized by the presence of osteochondromas located on the long bones and axial skeleton. Patients present with growth disturbances and angular deformities of the long bones as well as limited motion of affected joints. Forearm involvement is found in a considerable number of patients and may vary from the presence of a simple osteochondroma to severe forearm deformities and radial head dislocation. Patients encounter a variety of problems and symptoms e.g., pain, functional impairment, loss of strength and cosmetic concerns. Several surgical procedures are offered from excision of symptomatic osteochondromas to challenging reconstructions of forearm deformities. We describe visualizing, planning and treating these forearm deformities in MO and, in particular, a detailed account of the surgical correction of Masada type I and Masada type II MO forearm deformities.

## Introduction

Multiple osteochondromas (MO), also known as multiple hereditary exostoses (MHE), are disorder of endochondral bone growth producing abnormal metaphyseal bony prominences capped with cartilage. It is accompanied by defective metaphyseal remodelling and asymmetrical retardation of longitudinal bone growth [1]. MO is a rare, monogenetic, autosomal dominant disorder with an estimated prevalence of 1: 50,000 according to the older literature and 1:20,000–30,000 in more recent publications for the Dutch population [2, 3]. It is caused by loss of function mutations in either the exostosin-1 (EXT1 on chromosome 8) or exostosin-2 (EXT2 on chromosome 11) gene [4]. EXT1 and EXT2 mutations are found in over 90 % of all MO cases [5]. Whilst in 10 % of cases no EXT1 or EXT2 gene mutation is found, a third EXT loci has not been identified. A family history of MO exists in approximately 70–80 % of affected individuals, whereas 20–30 % of the cases are spontaneous mutations [3].

The forearm is involved in MO often, and osteochondromas are found most notably in the distal radius and ulna. Deformities of the forearm are reported in approximately 40–80 % of the patients and can be unilateral or bilateral, whereas one forearm is usually more severely affected than the other [1, 6–9]. Wrist osteochondromas and the developmental deformity give rise to complaints of pain and or progressive limitation of forearm rotation during growth. It has been suggested that the severity of forearm deformity correlates with overall disease severity and the risk of malignant degeneration [8].

The most common forearm deformities are:

1. a combination of relative shortening of either (usually the ulna) or both forearms
2. bowing of either one or both forearm bones
3. increased ulnar tilt of the distal radial epiphysis
4. ulnar deviation of the hand
5. progressive ulnar translocation of the carpus and
6. dislocation of the radial head [1, 6, 10–13].

**Fig. 1:**
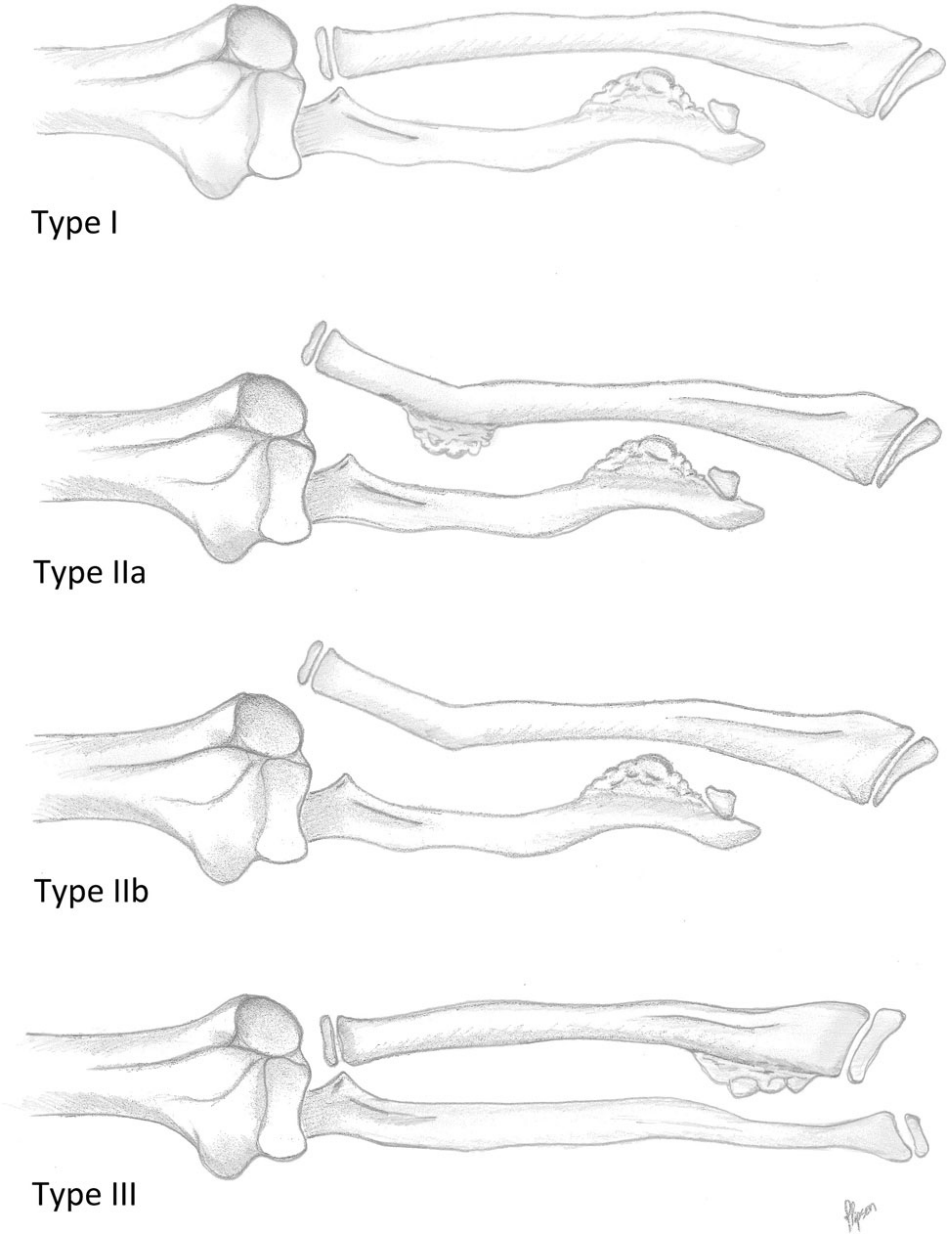
Schematic drawing of the Masada classification for forearm deformity in patients with MO [drawing by M.F., modified after 8]

The different deformities of the forearm are often classified according to the Masada deformity scale [11]; (Fig. 1). The treatment of these forearm deformities is difficult, and there is no consensus to overall management.

This report describes the strategies evolved by a MO-study group treating a large population of children with this disease in the Netherlands over the last 12 years.

## The Amsterdam MO database

In the Netherlands, the OLVG (Amsterdam) is regarded an expert centre for MO. Almost 600 patients with MO have been entered into a prospective database for various studies including a large series of 120 patients with forearm osteochondromas and deformities (140 forearms, surgically treated since 2002 by three surgeons AvdZ, JH and KM). Surgical procedures performed included excision of osteochondromas, ulnar lengthening, radial corrective osteotomy (proximal and distal), and excision of the radial head and neck (as a salvage procedure). A combination of procedures has been performed, e.g., corrective osteotomies of the radius (with plates) and lengthening of the ulna using hydroxyapatite-coated pins in a monolateral external fixator. All patients were counselled and provided informed consent to a structured treatment plan (Fig. 4). This is the strategy for treatment of forearm involvement in MO, and we describe the surgical technique for the extensive combined procedures performed in patients with severe forearm deformities and impaired function.

## Fundamentals of MO in the forearm

The Masada classification for forearm deformity from MO has been used since 1989 [11]. The classification is based on the morphological characteristics of the deformity on plain radiographs (Fig. 1). Three types are identified:

- *Type I* The main osteochondroma formation is located in the distal portion of the ulna. The ulna is shortened, and there is bowing of the radius. However, the radial head is not dislocated (this is the most common type in 55–61 % of cases).
- *Type II* In addition to ulnar shortening, the radial head is dislocated (22–33 % of patients). Bowing of the radius is less pronounced than in type I, and this could be an effect of the dislocation. In *subtype IIA*, the radial head is dislocated because of an additional osteochondroma at the proximal metaphysis of the radius. In *subtype IIB*, whilst there are osteochondromas at the distal ulna, there are none detectable in this region. Dislocation of the radial head leads to rotational impairment of pronation in general.
- *Type III* The main osteochondroma formation is in the metaphysis of the distal radius, and there is relative shortening of the radius.

According to Masada et al. [11], this classification indicates both the severity of the forearm deformity and the functional disabilities. Forearm rotation is most severely impaired in type I, whereas elbow motion is normal. Type II shows restriction of both elbow movement and forearm rotation. Radial deviation of the wrist is severely restricted in both subtypes. Type III retains most forearm and elbow movement, but ulnar deviation of the wrist is restricted and painful often.

## Diagnosis and imaging

**Fig. 2:**
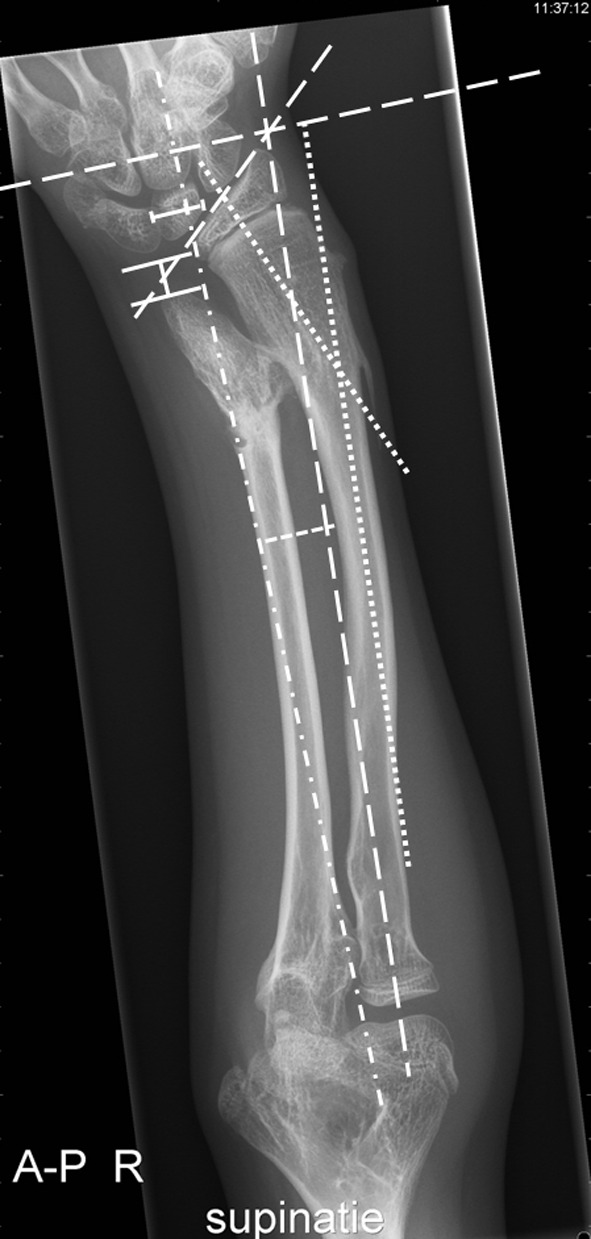
AP X-ray of the right forearm in a 14-year-old patient from the OLVG MO forearm database with Masada type I deformity: measurement of the RAA, CS, UV, RB and CORA [13]. *Dashed dotted lines* radial articular angle (RAA) between (1) a line perpendicular to a line that bisects the head of the radius and passes through the radial edge of the distal radial epiphysis and (2) a line along the articular surface of the distal radius. Normal values are defined between 15° and 30° by Fogel et al. [1]. Value measured: 43.1°.  Carpal slip (CS) percentage of the lunate surface in contact with the radius, as limited by the axial line drawn from the ulnar edge of the radial head through the ulnar edge of the radial epiphysis. This line normally bisects the lunate. Normal values for CS are >50 %. Value measured: 38 %.  Ulnar variance (UV) distance between the distal end of the ulna to the ulnar border of the distal radial epiphysis measured along the axial line. Normal values <15 mm. Value measured: 8.8 mm. *Dashed lines* radial bowing (RB) greatest distance between the radial diaphysis and the axial line. Normal values are defined as <12 mm. Value measured: 17.2 mm. *Dotted lines* centre of rotation of angulation (CORA) the intersection of the proximal axis and distal axis of a deformed bone

Anteroposterior radiographs of the entire forearm in full supination and pronation, completed with a lateral view are necessary to:

- visualize the presence of symptomatic and function-limiting osteochondromas
- to define the deformities in both forearm bones
- image all four joints (elbow, wrist, distal- and proximal radioulnar joints)
- to determine the centre of rotation and angulation (CORA) in case osteotomies have to be planned
- to locate the most appropriate site for ulnar lengthening (Fig. 2).

PA or AP radiographs can be obtained with the arm placed on the imaging plate with the shoulder at 90° of abduction and the elbow at 90° of flexion and for as far as is tolerable within patients’ range of motion. The beam is orthogonally directed towards the forearm in neutral position in the PA direction. Several angles and other variables can be measured on the radiographs and form the basis for follow-up during growth or outcome after forearm reconstruction. The two most important radiographic measurements are the radial articular angle (RAA) and the carpal slip (CS). These measurements are used frequently in the assessment, classification and follow-up of forearm deformities and were first described by Fogel et al. [14]. The RAA is the angle between two lines: one along the articular surface of the radius and the other perpendicular to a line from the centre of the radial head to the radial edge of the radial epiphysis. The normal value of the RAA is between 15 and 30° (Fig. 2) [14, 15]. The CS is measured as the percentage of the lunate in contact with the joint surface of the distal radius, using a line drawn from the centre of the olecranon through the ulnar edge of the radial epiphysis. This line bisects the lunate normally which makes the CS abnormal if ulnar displacement of the lunate is more than 50 % [11, 16]. These measurements are of interest since both have implications for loads across the lunate. An increase of CS or a decrease of RAA will intensify the radio-carpal force, with potential links to arthritic changes, pain and loss of range of motion. MRI can provide additional information on the extent of osteochondromas, the width of the cartilage cap, and the relation to or involvement of neurovascular structures. It can show changes in the ligamentous structures in both the joints and the intraosseous membrane, visualize the pathoanatomy of the soft tissues in chronic radial head dislocations and display the organization of the distal radioulnar joint (DRUJ) in complex ulnar-minus variants.

CT scans display the distorted anatomy at different angles as well in 3D and are useful in surgical planning. These images are very informative and helpful during the counselling of the patient and their relatives. CT images give more insight into joint anatomy and may visualize (early) degenerative changes. Special scanning protocols with the neighbouring joints can be used for computerized planning templates.

## Should MO forearm deformities be operated on and, if so, when?

Despite the 25 years following the original paper by Masada, the optimal treatment for an individual patient with MO of the forearm is unresolved. Published studies in the past are critiqued for being poorly designed, being retrospective case series with short follow-up, lacking detailed descriptions of the types of deformations (Masada-types) treated, using different indications for surgery (if mentioned at all), using different surgical procedures, and lacking outcome measurements. A lack of information on external validity items and well-defined outcomes can lead to difficulties in extrapolating the results of a study to other MO-patients with forearm deformities. Although the review performed on the outcome of surgical treatment by this study group showed an overall benefit from surgery, there was no control group for a comparison to the natural history of the condition [17]. Data on this are lacking and as such a comparison to those treated difficult [18].

The age reconstructive surgery should be performed in children is debatable. There are two opposing opinions in the literature. Several authors recommend early surgical intervention, but in their report the mean follow-up is short and appears insufficient to assess for recurrence [10]. The second view, represented by Akita et al., proposes a less aggressive approach involving surgical interventions towards the end of the growth spurt. The longer-term follow-up (13 years) of their study revealed recurrence in children who underwent surgery too early [1]. We share the same experience; we counsel and follow the patients, if possible, until the age of 13–15 years and recommend intervention then and as one correction.

As with Litzelmann et al., we see radial head instability to be a major prognostic factor as this is associated with symptoms frequently. If progressive radial head dislocations are detected, ulnar lengthening by callus distraction and corrective osteotomy of the radius should be considered early. This is done to avoid development of pain or restriction of pronation and supination at the elbow level [10, 19]. The maintenance of a reduced radial head following these “levelling or rebalancing procedures” is, however, uncertain and a second (salvage) procedure necessary at a later stage.

## MO-study group protocol

Specific indications for surgery are determined. Pain due to impingement of an osteochondroma, restriction of motion, functional deficits, loss of strength, severity of deformity and/or dislocation of the radial head are recorded. The Masada classification still forms the basis for surgery.

1. Patients with Masada type I deformity with mild radial bowing and mild symptoms will be counselled for conservative treatment and receive yearly follow-up. In patients with large osteochondromas which are painful despite minimal functional impairment, elective removal of the symptomatic osteochondroma is recommended as are those which give rise to gradual erosion and deformity of the adjacent bone and limited rotation and pain.
2. Patients with advanced deformity and major functional impairment will be counselled to wait until the age of 13. Only if there is a large impact on daily living would a corrective osteotomy of the radius and lengthening with monolateral fixation, even at young age, be performed and with yearly follow-up. In patients with Masada type IIA deformity, removal of the proximal osteochondroma (as they are painful) and, in type IIB, removal of the ulnar osteochondroma is recommended. An expectant approach is carried out for Masada type II problems if symptoms are mild and the radial head is stable. If advanced functional impairment as well as instability and pain is present, we either plan a 2-stage procedure (first levelling of the forearm bones and, later, a radial head resection—rarely) or we advocate a complex correction with indirect reduction of the radial head using a ring fixator [20, 21].
3. In patients with type III deformity, we offer to remove the painful osteochondroma but counsel most patients to wait until growth is completed. Some patients in this group need treatment for their positive ulnar variance, usually by ulnar shortening osteotomy.

## Correction in advanced MO Masada type I or levelling procedure in Masada IIB

**Fig. 3:**
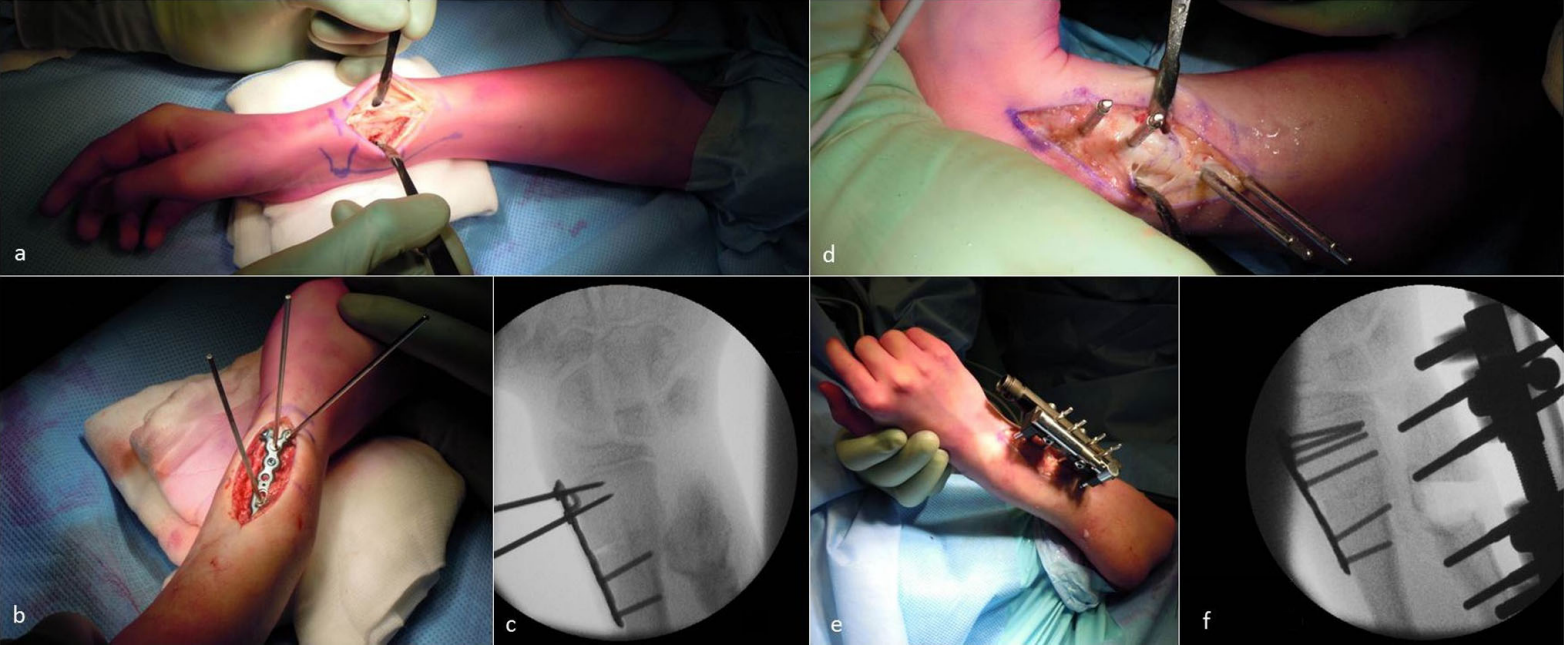
Intraoperative images of a corrective osteotomy and ulnar lengthening fixator application at the right forearm with a Masada type I deformity (patient from Fig. 2). **a** clinical image of the right forearm, after marking of the CORA the osteotomy site at the radius is exposed; **b** a closing wedge osteotomy is performed (15°) using FFS (Orthofix^®^) for temporary fixation and a low profile plate (Medartis^®^); **c** fluoroscopic image showing correction of the radius and good positioning of the plate proximal to the growth plate; **d** via a direct ulnar approach 4 fixator pins are placed into the distal ulna, note the use of different angles both in rotation and abduction of the pins in order to correct the deformity of the ulna by pin placement; **e** the monolateral lengthening fixator is mounted, both radius and ulna are straightened; **f** fluoroscopy shows the radius osteotomy and a nice alignment of the ulna (under test distraction)

Thorough counselling is recommended prior to a full treatment plan being advocated. These reconstructive procedures are elective, and a clinical benefit remains to be proven. In large deformities, we start with a corrective osteotomy of the radius (Fig. 3), but in those with a mild deformity of the radius, ulnar lengthening (often paired with slight translation of the distal ulnar segment to avoid impingement in the DRUJ) is possible. If using monolateral fixators, it is important to correct acutely the concomitant deformation of the ulna (bowing, rotation and adduction of the distal ulna due to tethering effect of the soft tissues and disorientation of the distal ulnar growth plate) through appropriate placement of the fixator pins (Fig. 3). The osteotomy is performed percutaneously using a sharp drill through a drill-guide with normal saline cooling and completion of the osteotomy with a sharp chisel. Care must be taken not to split the bone, especially in the area where an osteochondroma has been removed as the bone can be brittle. An intraoperative test for distraction is performed to ensure completion of the osteotomy. Distraction for gradual lengthening starts after a latency period of a minimum of 5 days post-operatively with 3 × 0.25 mm lengthening per day until the target length (levelling) is reached.

### Preoperative planning and the surgical technique

**Fig. 4:**
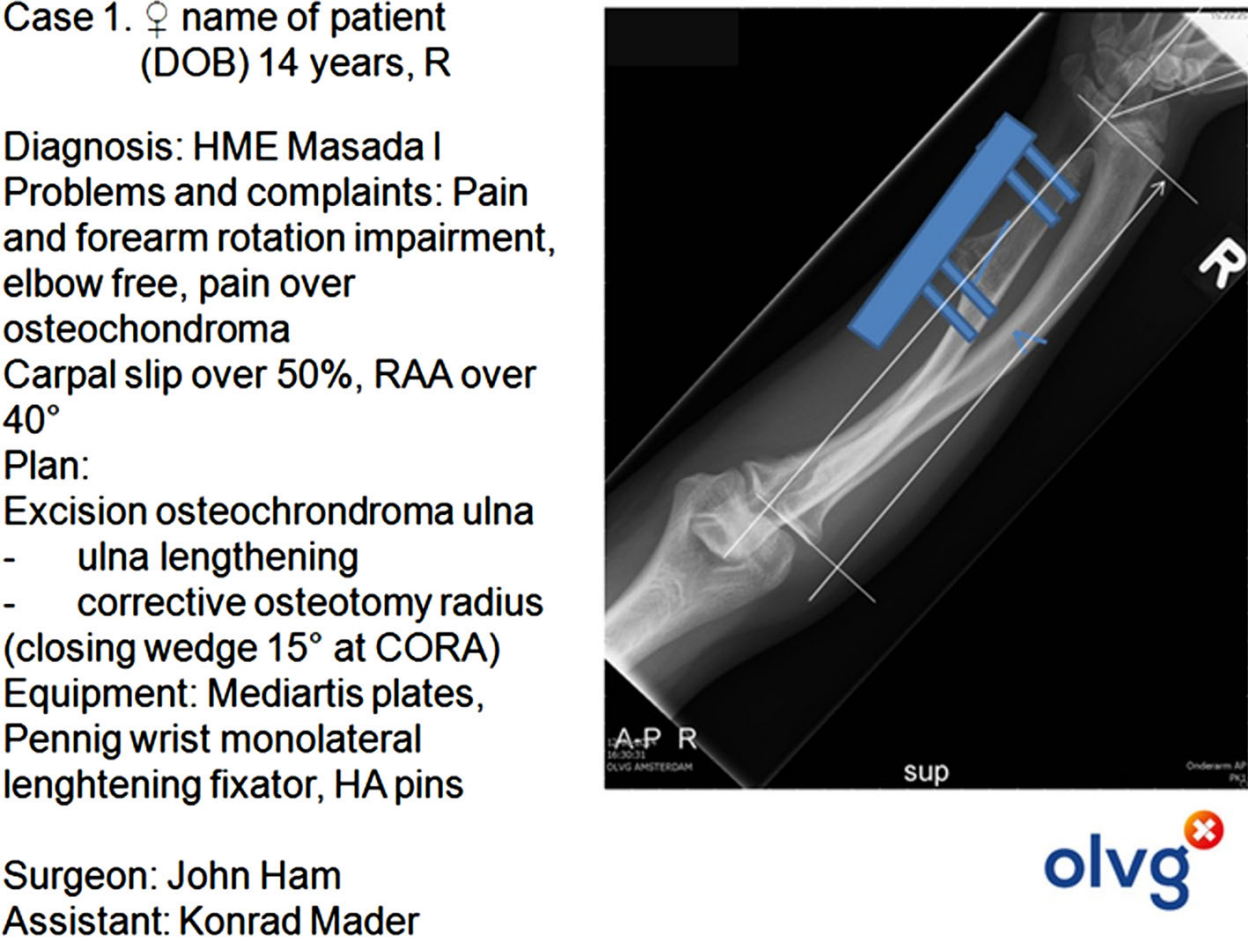
Printout of a structured treatment plan for a 14-year-old patient with MO Masada I

All surgeries for the past 14 years were performed with prior counselling of the patient and parent(s) and with full informed consent; special emphasis was made to consider the relative merits of operative and conservative treatment (Fig. 4). In all cases, the main indication for surgery was pain and/or functional impairment, documented as an active and passive motion of both the wrist and elbow using the neutral—0—method. Preoperative planning was an essential and integral part of deformity correction surgery. In all cases forearm radiographs, and later CT scan reconstruction images, were used for conceptual drawings of surgery and to formulate a structured treatment plan [21]; key information on the medical condition and treatment history, symptoms and functional impairment, the problems (deformity) to be addressed, the planning method used (conventional versus computerized), the surgical procedure (with the different operative steps), the equipment needed and potential obstacles during or after the surgery (Fig. 4). A basic stepwise approach was used for analysing the deformity. In cases of combined procedures (i.e., removal of one or more osteochondromas at the distal radius or ulna in conjunction with a corrective osteotomy of the radius and monolateral lengthening of the ulna), the osteochondromas were removed first followed by an osteotomy of the radius at the CORA with the application of a monolateral lengthening fixator. In all cases, hydroxyapatite screws (Orthofix^®^) were used and the Pennig monolateral lengthener (Orthofix^®^).

All operations were performed by consultant orthopaedic surgeons, with a minimum of two surgeons assisting each other (JH, AvdZ, KM). Full general anaesthesia and a tourniquet (200 mmHg for a maximum of 90 min) and hand table were used. Fluoroscopy was used to mark the deformity, the CORA at the radius, the osteotomy level and pin insertion areas in the ulna.

A direct radial approach to the radius over the CORA was performed with the superficial branch of the radial nerve visualized and protected. In most cases, a closing wedge osteotomy was performed using the Fragment Fixation System (FFS; Orthofix^®^) for temporary fixation and a low profile plate (Depuy Synthes^®^ or Medartis^®^) for definitive fixation. Great care was taken not to damage the distal growth plate of the radius (Fig. 3). From the preoperative planning, four fixator pins (typically 80 mm length with 20 mm thread length (conical blunt-tipped and hydroxyapatite coated, Orthofix^®^) were placed into the distal ulna through stab incisions. Using special screw and drill guides and with predrilling and cooling, different angles of pin placement with regard to rotation and abduction allowed for pre-emptive correction of the deformity of the ulna (Fig. 4). Meticulous care was taken for good purchase and bicortical penetration of all pins, which was difficult due to previous or simultaneous resection of osteochondromas in the area. The osteotomy was performed using a sharp drill and a chisel whilst preserving the periosteum. The monolateral lengthening fixator was mounted, the acute re-alignment of the ulna was documented, and a test distraction performed (Fig. 3). Wounds were closed using resorbable subcutaneous and skin sutures. A posterior backslab cast with the wrist in neutral position was applied. After a waiting period of 5 days (usually), lengthening was performed following a written protocol (0.25 mm × 3 per day) until the required length was reached. The fixator was removed under light anaesthesia when callus maturation was documented by standard radiographs (three cortices visible).

## Outcome

From a systematic review (unpublished) of 16 studies, both the ulnar lengthening procedure and a radial correction with ulnar lengthening improved clinical and radiographic parameters significantly [17]. All clinical and radiographic parameters of patients with MO of the forearm were worse than in healthy individuals before surgery; patients with worse baseline parameters benefited the most from surgery.

Ulnar lengthening with or without excision of osteochondroma(s) improved range of motion of the forearm and elbow, wrist radial deviation, the forearm bone length discrepancy (levelling), radial bowing, radial articular angle and carpal slip of the forearm. A radial osteotomy and or radial stapling with or without ulnar lengthening or excision of osteochondromas had the same improvement across the same parameters; there were fewer radial head dislocations after the procedure(s). Simple removal of osteochondromas seemed to improve the range of motion but without improvement of radiographic parameters (RAA, CS).

Post-surgical patient-reported outcomes were improved, but the complication rate of ulnar lengthening was high. Radial osteotomy and or radial stapling with or without excision of osteochondroma showed a lower complication rate but a higher risk of recurrence.

The internal and external validity of the included studies in the systematic review was low as important criteria were missing on different items. A lack of information on internal validity items, such as the representativeness of the study group, duration of follow-up, blind assessment of the outcome and adjustment for other factors could have led to invalid results. This demonstrated that the results of surgical management for forearm deformities in patients with multiple osteochondromas are, as yet, not yet clear.

A lack of evidence as to which procedure gives optimal results in forearm MO long-term prompted a review of our own data before starting a prospective trial. From retrospective studies in which the first 94 patients (125 forearms) operated on were included, we concluded that significant improvements were made in pain complaints and range of motion by excision of osteochondromas or corrective procedures in patients with forearm deformities in MO. Following the treatment protocol described, significant improvements were made for Masada type 0 (no deformities but only symptomatic osteochondroma(s) resulting in pain or functional loss) and type I patients in the parameters of pronation, supination, dorsal extension, and radial deviation after 2, 5 and 10 years. Additionally, for Masada type 0 and I patients pain improved significantly after excision of osteochondromas. Compared to Masada type 0 and I, Masada type II, patients tend to have lower preoperative DASH scores, more severe functional impairment and have from aesthetic concerns.

## Conclusion

We present a summary of a treatment protocol and the basis for visualizing, planning and treating forearm deformities in MO. We have described our current method of surgical correction of Masada type I and the levelling procedure in Masada type II MO forearm deformities. Whilst initial results are encouraging, more research is needed for prognostic variables that might influence outcome and patient satisfaction in surgery for forearm abnormalities from multiple osteochondromas.
